# Supplementary material for: Molecular Dynamics Simulation of Polymer Nanocomposites with Supramolecular Network Constructed via Functionalized Polymer End-Grafted Nanoparticles
Source: Polymers (Basel). 2023 Jul 31;15(15):3259. doi: 10.3390/polym15153259 (PMC10422474; doi:10.3390/polym15153259)
Supplement: Supplementary file 1 [file polymers-15-03259-s001.zip › polymers-2512505-supplementary.pdf]

Supplementary Materials

Molecular Dynamics Simulation of Polymer Nanocomposites with Supramolecular Network Constructed via Functionalized Polymer End-Grafted Nanoparticles

Guanyi Hou [1,*], Runhan Ren [1], Wei Shang [1], Yunxuan Weng [1,*] and Jun Liu [2]

[1] College of Chemistry and Materials Engineering, Beijing Technology and Business University, Beijing 100048, China; renrunhan@163.com (R.R.); herzflut@foxmail.com (W.S.)
[2] Center of Advanced Elastomer Materials, Beijing University of Chemical Technology, Beijing 100029, China; lj200321039@163.com
* Correspondence: hgy1939916@163.com (G.H.); wyxuan@th.btbu.edu.cn (Y.W.)

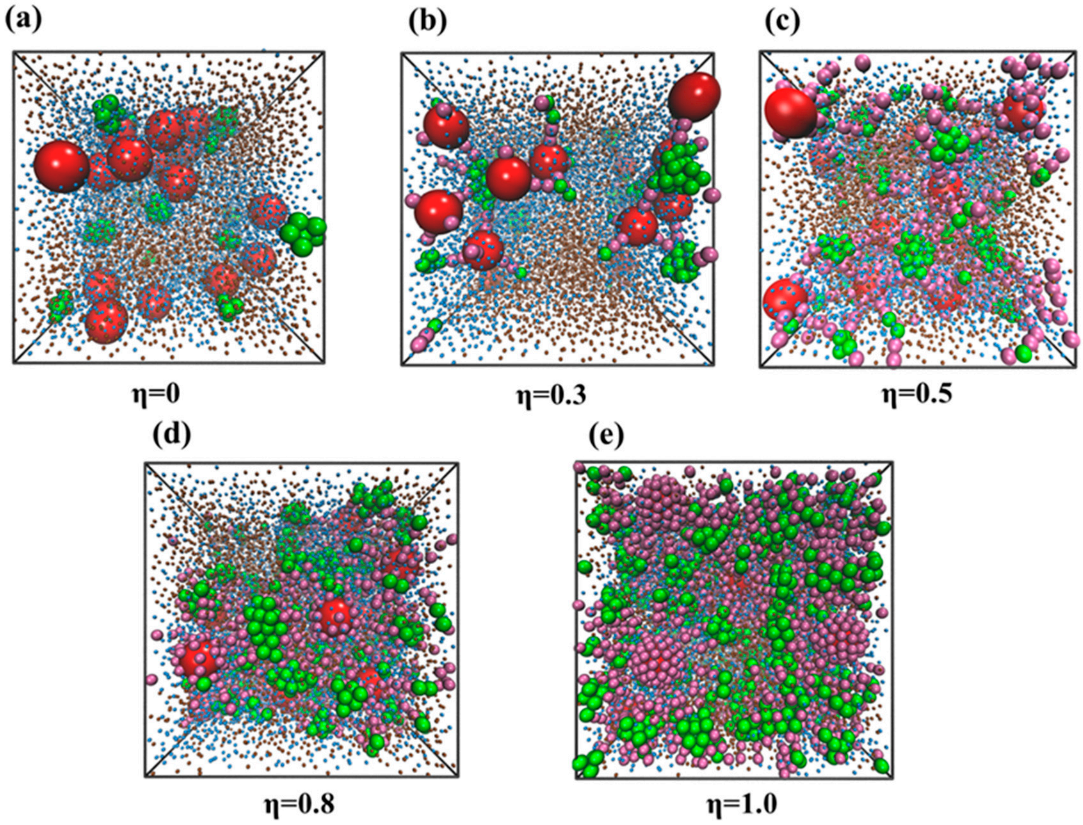

Figure S1. the snapshot of PNCs with different η, where: (a) η = 0; (b) η = 0.3; (c) η = 0.5; (d) η = 0.8; (e) η = 1.0.

Table S1. the mean-square root radius of gyration of modified-NP (R_g)

System	Mean-square root radius of gyration of modified-NP (σ)
η = 0	2.0
η = 0.3	5.07
η = 0.5	5.51
η = 0.8	5.79
η = 1.0	5.96

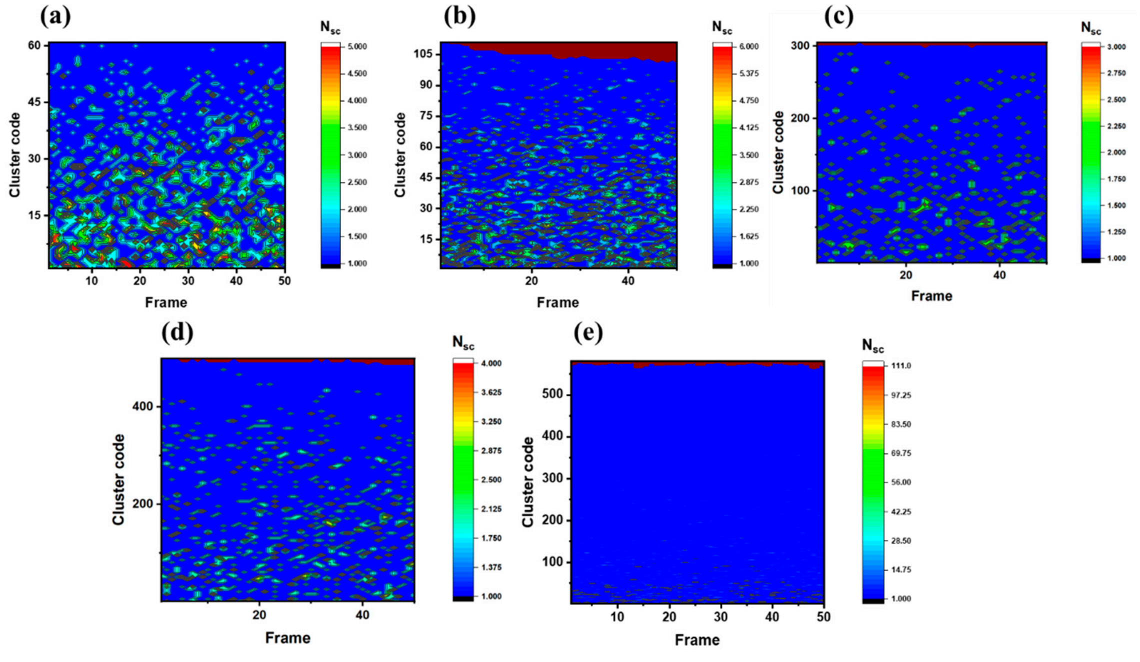

Figure S2. The size and distribution of clusters, which are formed by the modified group, with various sphericity (η) where (a) η = 0; (b) η = 0.3; (c) η = 0.5; (d) η = 0.8 and (e) η = 1.0.

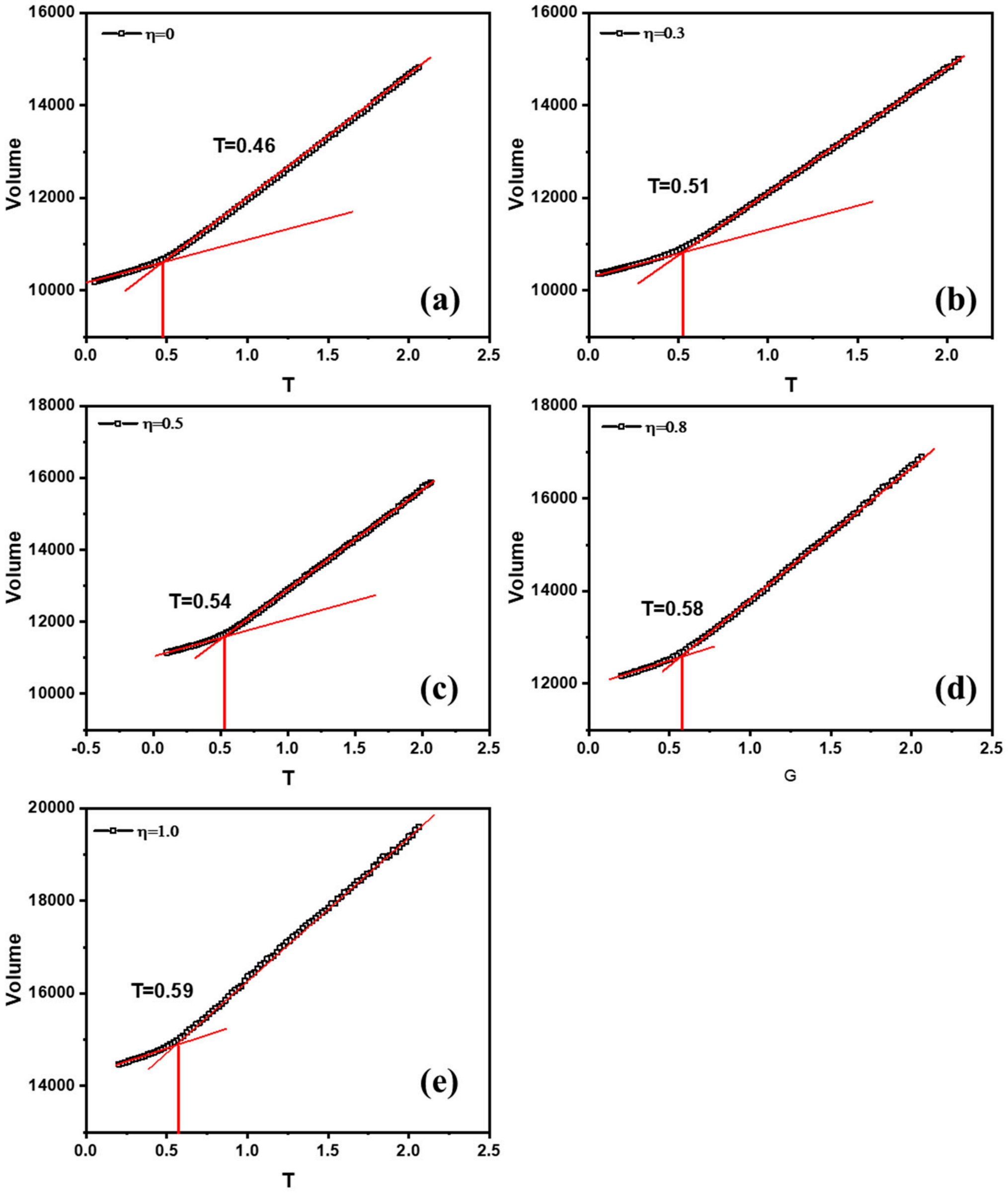

Figure S3. The glass transition temperature (T_g) obtained by the specific volume versus the temperature for different η, where (a) η = 0; (b) η = 0.3; (c) η = 0.5; (d) η = 0.8 and (e) η = 1.0.
